# Supplementary material for: Plant Reproductive Success Mediated by Nectar Offered to Pollinators and Defensive Ants in Terrestrial Bromeliaceae
Source: Plants (Basel). 2024 Feb 8;13(4):493. doi: 10.3390/plants13040493 (PMC10891524; doi:10.3390/plants13040493)
Supplement: Supplementary file 1 [file plants-13-00493-s001.zip › plants-2807695-supplementary.pdf]

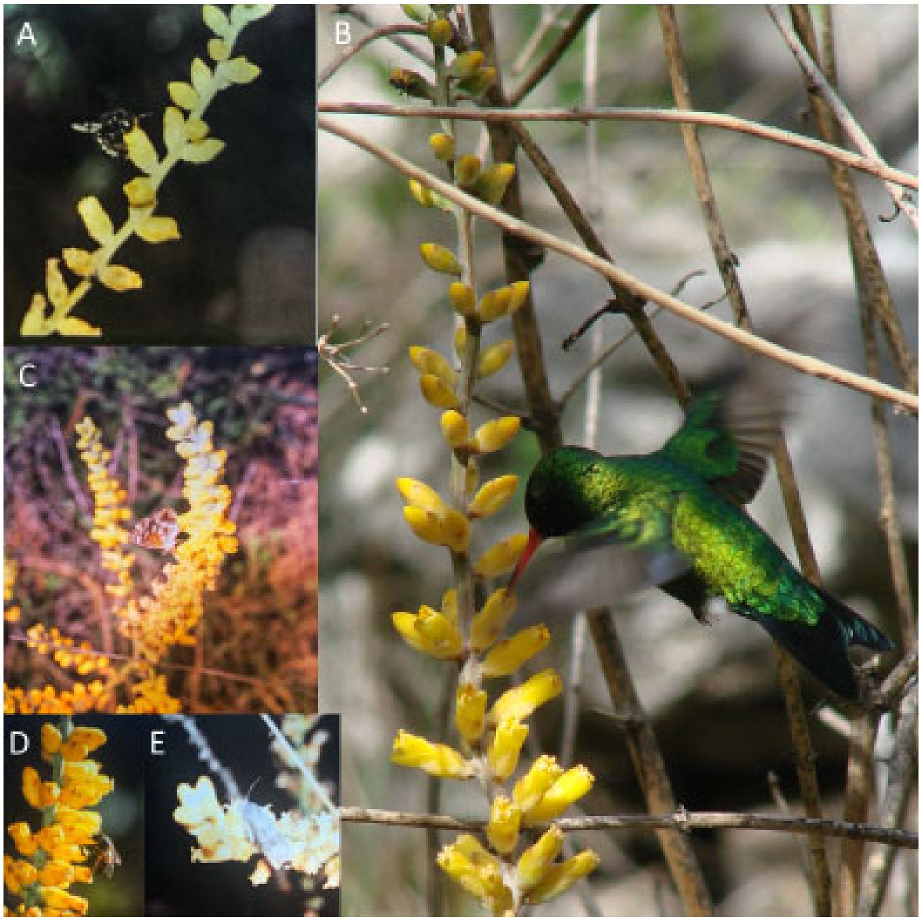

Figure S1. Pollinators visiting flowers for floral nectar and or pollen in *Dyckia floribunda*. (**A**). *Xylocopa* sp. (**B**). *Chlorostilbon lucidus*. (**C**). *Vanessa* sp. (**D**). *Apis mellifera*. (**E**). A moth, representative of Noctuideae.
